# Evaluation of serum iron overload, AST:ALT ratio and log_10_ferritin:AST ratio among schizophrenia patients in the Kumasi Metropolis, Ghana: a case–control study

**DOI:** 10.1186/s13104-019-4847-2

**Published:** 2019-12-12

**Authors:** W. K. B. A. Owiredu, Peter Kojo Brenya, Yaw Osei, Edwin Ferguson Laing, Clement Opoku Okrah, Christian Obirikorang, Enoch Odame Anto, Emmanuel Acheampong, Sampson Donkor

**Affiliations:** 1 Department of Molecular Medicine, KNUST School of Medicine and Dentistry, Kwame Nkrumah University of Science and Technology (KNUST), Kumasi, Ghana; 2 School of Medical and Health Sciences, Edith Cowan University (ECU), Joondalup, Perth-WA Australia; 3 Department of Physician Assistance, Faculty of Health and Allied Sciences, Garden City University College (GCUC), Kenyasi, Kumasi, Ghana; 4 Department of Medical Laboratory Technology, Faculty of Allied Health Science, KNUST, Kumasi, Ghana

**Keywords:** Iron overload, Schizophrenia, AST:ALT ratio, log_10_ferritin:AST

## Abstract

**Objective:**

The association between unbalanced iron indices and the conditions of schizophrenia are not well understood. Liver dysfunction which has been linked to iron metabolism might be a contributing factor. This case–control study evaluated serum iron indices and liver function in treatment-naïve schizophrenia patients and those already on treatment at the Psychiatric Department of the Komfo Anokye Teaching Hospital (KATH), Kumasi-Ghana.

**Results:**

The mean age of the respondents was 39.6 ± 0.8 years. Increased levels of serum iron, TS, AST, ALT and AST:ALT ratio and lower levels of UIBC, TIBC, Transferrin, and log Ferritin:AST ratio levels were observed among the treatment-naïve group compared to the control. The treatment-naïve and treatment groups showed significantly higher serum AST:ALT ratio, and lower log_10_ferrtin:AST ratio than the healthy controls. There was a significant correlation between log_10_ferritin and AST, and log_10_ferritin and GGT in both treatments (r = 0.343; p = 0.003, and r = 0.502; p = 0.001 respectively) and treatment-naïve groups (r = 0.348; p = 0.002, and r = 0.614; p < 0.001 respectively). Percentage transferrin saturation correlated significantly with GGT only, in the treatment-naïve group (r = 0.667; p < 0.001), and ALT and GGT in the treatment group (r = 0.252; p = 0.030 and r = 0.646; p = 0.014 respectively).

## Introduction

According to the World Health Organization (WHO) [1], mental disorders account for five of the ten leading causes of disability and premature death worldwide, of which neuropsychiatric condition accounts for 13% of the total disability adjusted life years [1]. A prevalence based rate report from the World Mental Health Survey 2004, undertaken by WHO in Ghana estimated that, 13% of the adult population suffer from some form of mental disorder [2].

Studies in Ghana have indicated that schizophrenia is the leading psychiatric condition [3, 4]. Owiredu et al. [5] in their study in Ghana reported a prevalence of 59% for schizophrenia compared to other psychiatric conditions [5]. Serious mental illness is accompanied by higher morbidity and mortality rates of chronic diseases such as lung disease, diabetes and liver problems [6]. A previous study found that liver dysfunction is particularly elevated in adults with mental illness [7]. Iron overload has been reported to be significantly associated with liver damage [8].

Iron overload (IO) has been reported to be associated with parenchymal and organ dysfunction following abnormal deposition of tissue iron [9, 10]. Dangers of IO in several disease conditions over the years including coronary disease [10–12], diabetes mellitus [10, 13], sexual impotence [9, 10], cerebral ischemic disease [10, 14] and neurologic or psychiatric alterations [10, 15] has been noted.

The manifestations of neuropsychiatric effects of chronic iron overload have been observed in depressives, memory loss, paranoia, visual hallucination, lethargy, disorientation, dementia, and anxiety symptoms [16, 17]. However, the pathophysiology of iron metabolism in psychiatric illnesses still remains unclear, albeit several studies have suggested a possible link between serum iron and some variables in psychiatric conditions [16, 18].

There is dearth of data on serum iron indices among psychiatric patients in Ghana, and the association between iron overload and primary psychiatric illness. Also, little is known about the liver function of psychiatric patients in Ghana. This study therefore evaluated serum iron indices and liver function among schizophrenia patients in a Ghanaian population.

## Main text

### Methods

#### Study population and setting

This case–control study was carried out at the Psychiatric department of the Komfo Anokye Teaching Hospital, Kumasi-Ghana. Qualifying patients attending the psychiatric department were recruited into the study using International classification of Diseases (ICD-10). Healthy control participants were recruited from a keep fit club. A total of 200 participants comprising 75 treatment-naïve schizophrenia patients, 75 schizophrenia patients on treatment and 50 healthy controls were recruited via purposive sampling.

#### Ethical consideration

The study was approved by the Committee on Human Research, Publications and Ethics (CHRPE), and the Research and Development Unit of the Komfo Anokye Teaching Hospital (KATH). Written informed consent was obtained from each participant through their legally authorised family members before enrolment into the study. All data obtained from participants was held under strict confidentiality.

#### Inclusion criteria

Newly diagnosed naïve schizophrenia patients and those already on any form of antipsychotic, such as olanzapine, haloperidol, risperidone etc., were included in the study. Age and sex-matched healthy individuals who were not on any medication (antibiotics, vitamins supplement), non-alcoholic, Tuberculosis free, HIV free, Hepatitis B virus free, and were not presenting any signs of chronic illness were included in the study. All respondents were ≥ 18 years.

#### Exclusion criteria

Schizophrenia patients already on iron therapy, non-steroidal anti-inflammatory agents, antacids, alcohol consumption, pain killers such as paracetamol etc., multiple blood transfusions and androgen/oestrogen therapy were all excluded from the study.

#### Questionnaire administration and sample collection

A validated questionnaire was administered to all respondents by qualified nurses to collect demographic data including age and gender. About 4 ml of venous blood sample was collected from the antecubital fosa of the study participants after an overnight fast, and sera were obtained after centrifugation. Assay parameters included: serum iron, unsaturated iron binding capacity (UIBC), total iron binding capacity (TIBC), transferrin, ferritin, percentage transferrin saturation, aspartate aminotransferase (AST), gamma glutamyl transferase (GGT) and alanine aminotransferase (ALT). AST, GGT and ALT were performed on a fully automated Mindray *BS*-380 auto-analyzer (Shenzhen Mindray Bio-medical electronics Co., Ltd, China). Ferritin was performed on Mindray^®^ microplate reader *MR* 96 A. The assay for UIBC and iron were performed on the Mindray *BA*-88A Biochemistry auto-analyzer. Transferrin concentration was estimated according to Vernet [19] and Gambino et al. [20] equation.

#### Statistical analysis

Results are presented as mean ± SD. Analysis of variance (ANOVA) coupled with Tukey’s post hoc multiple comparisons was used to compare more than two means of continuous variables. Unpaired t-test was used to compare the means of two continuous variables. Chi-square test was used to assess the associations between categorized variables. Partial Pearson correlation was performed to test for associations between iron indices and liver function markers. A p-value < 0.05 was considered statistically significant for all analyzed data. Statistical analyses were performed using GraphPad Prism 7.

### Results

Table 1 shows demographic characteristics of age and gender of the study participants. Out of the total 200 participants, 104 (52%) were females and 96 (48%) were males. The mean age of the study population was 39.6 years. There was no significant difference between the mean age of the control group compared to the treatment (p = 0.4654) and treatment-naïve group (p = 0.4120).

**Table 1:** Demographic characteristics of the study participants

Variables	Total (n = 200)	Control (n = 50)	T (n = 75)	^‡^*p*-value	TN (n = 75)	^*#*^*p*-value
Age (years)	39.6 ± 0.8	40.5 ± 1.4	39.0 ± 1.3	0.4654	38.9 ± 1.4	0.412
Gender				0.4723		0.7169
Male	96 (48.0%)	26 (52.0%)	34 (45.3%)		36 (48.0%)	
Female	104 (52.0%)	24 (48.0%)	41 (54.7%)		39 (52.0%)	

Values are presented as mean ± standard deviation (SD) and frequency (proportion) where appropriate

*T* treatment, *TN* treatment naïve

^‡^p-value (comparison between control and treatment)

^#^p-value (comparison between control and treatment naïve)

Serum iron concentration and percentage transferrin saturation were significantly higher in treatment-naïve psychiatric patients compared to the control (p < 0.0001). Mean levels of transferrin, UIBC and TIBC was significantly lower in treatment-naïve psychiatric patients compared to the control (p < 0.0001). The levels of serum ferritin was highest among the treatment-naïve group, followed by the treatment group, and did not differ significantly compared to the control. Generally, the treatment-naïve group recorded the highest mean AST, ALT and GGT levels, followed by the treatment group. There was a statistically significant difference between the AST and GGT levels of the treatment-naïve group and that of the control group (p = 0.0035 and 0.0418 respectively). Both the treatment-naïve and treatment groups had higher mean AST:ALT ratio (1.61; p = 0.0004 and 1.50; p = 0.0282 respectively) than the control group (1.29). However, mean log ferritin:AST ratio decreased significantly from the control (0.096) to treatment group (0.084, p = 0.0448) and then to the treatment-naïve group (0.080; p = 0.0113) (Table 2).

**Table 2:** Biochemical profile of the study participants

Variables	Total (n = 200)	Control (N = 50)	T (n = 75)	^‡^p-value	TN (N = 75)	^#^p-value
Markers of iron overload						
Serum iron (µmol/l)	37.2 ± 1.1	31.3 ± 1.8	33.5 ± 1.4	0.3519	44.9 ± 1.8	< 0.0001
Serum UIBC (µmol/l)	60.9 ± 1.6	69.6 ± 2.1	66.2 ± 2.5	0.2846	40.0 ± 2.5	< 0.0001
Serum TIBC (µmol/l)	95.2 ± 2.4	100.9 ± 3.7	99.6 ± 3.4	0.5374	84.9 ± 3.1	< 0.0001
Serum ferritin (ng/ml)	92.1 ± 5.1	81.6 ± 7.5	94.3 ± 8.2	0.2827	97.0 ± 10.9	0.2956
log_10_ (ferritin)	1.8 ± 0.0	1.8 ± 0.1	1.9 ± 0.0	0.5674	1.9 ± 0.0	0.3975
Serum transferrin (g/l)	3.8 ± 0.1	4.02 ± 0.1	3.9 ± 0.1	0.3761	3.4 ± 0.1	< 0.0001
% transferrin saturation	39.2 ± 0.8	31.01 ± 0.9	33.6 ± 1.0	0.3145	52.9 ± 1.6	< 0.0001
Markers of liver function						
Serum AST (U/l)	24.7 ± 0.9	20.9 ± 1.2	23.9 ± 1.2	0.0773	28.0 ± 1.8	0.0035
Serum ALT (U/l)	17.9 ± 0.6	17.8 ± 1.4	17.4 ± 0.8	0.8240	18.5 ± 1.1	0.6570
GGT (U/l)	29.3 ± 1.8	21.32 ± 1.3	29.7 ± 1.9	0.0503	36.98 ± 2.4	0.0418
AST:ALT ratio	1.5 ± 0.0	1.29 ± 0.0	1.50 ± 0.1	0.0282	1.61 ± 0.1	0.0004
log_10_ferrtin:AST ratio	0.087 ± 0.0	0.096 ± 0.0	0.084 ± 0.0	0.0448	0.080 ± 0.0	0.0113

Values are presented as mean ± standard deviation (SD) and frequency (proportion) where appropriate

*UIBC* unsaturated iron binding capacity, *TIBC* total iron binding capacity, *AST* aspartate aminotransferase, *ALT* alanine aminotransferase, *T* treatment, *GGT* gamma-glutamyl transferase, *TN* treatment naïve

^‡^p-value (comparison between control and treatment)

^#^p-value (comparison between control and treatment naïve)

Table 3 shows the biochemical profile of the study population stratified by gender. There was no statistical difference between any biochemical parameter and gender among the control group. Both males and females in the treatment and treatment-naïve groups reported higher mean levels of iron, transferrin saturation, ALT, GGT and AST but lower levels of UIBC rather than the control group. The male respondents of the treatment-naïve group showed significantly higher mean serum iron, UIBC and STfR (p < 0.0001 each), as well as higher TIBC (p = 0.0026) and log_10_ferritin (p = 0.0035) rather than their female counterparts. There was no significant difference among gender with respect to the liver function parameters, albeit the males showed higher mean levels of AST, ALT and GGT. With the exception of the comparable means of log_10_ferritn between the female treatment group and the female controls, all gender-matched comparisons across the various groups were statistically significant (p < 0.05).

**Tables 3:** Biochemical profile of study population in relation to gender

Parameter	Control		p-value	T		p-value	TN		p-value
	Males (n = 26)	Females (n = 24)		Male (n = 34)	Female (n = 41)		Male (n = 36)	Female (n = 39)	
Iron (µmol/l)	26.53 ± 2.8	25.66 ± 1.69	0.0921	^‡^36.33 ± 2.01^$^	^#^30.82 ± 1.99^$$^	0.0573	^‡^52.68 ± 2.75	^#^35.85 ± 1.89	< 0.0001
UIBC (µmol/l)	42.12 ± 4.40	39.76 ± 3.35	0.0604	^‡^26.46 ± 3.6^$^	^#^25.18 ± 2.99^$$^	0.5290	^‡^39.63 ± 4.02	^#^33.04 ± 5.34	< 0.0001
TIBC (µmol/l)	60.65 ± 5.7	59.03 ± 4.03	0.7190	^‡^67.14 ± 2.65^$^	^#^61.03 ± 3.01^$$^	0.7330	^‡^75.96 ± 2.87	^#^63.86 ± 2.61	0.0026
log_10_ferritin	1.72 ± 0.05	1.71 ± 0.07	0.0807	^‡^2.00 ± 0.04^$^	1.72 ± 0.06^$$^	0.0005	^‡^1.97 ± 0.05	^#^1.76 ± 0.05	0.0035
STfR (µmol/l)	2.29 ± 0. 18	2.35 ± 0.13	0.0899	^‡^4.12 ± 0.16^$^	^#^3.83 ± 0.21^$$^	0.2977	^‡^5.13 ± 0.23	^#^4.05 ± 0.12	< 0.0001
%T. saturation	44.88 ± 1.16	44.52 ± 2.64	0.9132	^‡^34.97 ± 1.7^$^	^#^32.02 ± 1.34^$$^	0.1092	^‡^63.56 ± 1.76	^#^58.74 ± 2.64	0.1188
Markers of liver function									
AST (U/l)	17.92 ± 1.80	17.17 ± 1.01	0.0512	^‡^23.03 ± 0.85^$^	^#^24.78 ± 2.00^$$^	0.4558	^‡^30.53 ± 3.21	^#^25.69 ± 1.66	0.1756
ALT (U/l)	15.50 ± 2.07	14.79 ± 1.63	0.0668	^‡^18.09 ± 1.17^$^	^#^16.88 ± 1.12^$$^	0.4593	^‡^19.31 ± 1.83	^#^17.82 ± 1.23	0.4967
GGT	21.10 ± 1.6	20.31 ± 1.4	0.0701	^‡^29.81 ± 1.9^$^	^#^28.12 ± 1.3^$$^	0.6311	^‡^36.81 ± 2.3	^#^35.21 ± 2.0	0.5571

Values are presented as mean ± standard deviation (SD)

*T* treatment, *TN* treatment naïve

^‡^p<0.05 for comparison between male control and male T or TN group)

^#^p < 0.05 for comparison between female control and female T or TN group

^$^p<0.05 for comparison between male T and male TN group

^$$^p<0.05 for comparison between male T male TN group

Additional file 1: Table S1 shows the association between iron indices and liver function parameters among both treatment and treatment-naïve schizophrenia patients. There was a positive and significant correlation between log_10_ferritin and AST, and log_10_ferritin and GGT in both treatments (r = 0.343; p = 0.003, and r = 0.502; p = 0.001 respectively) and treatment-naïve groups (r = 0.348; p = 0.002, and r = 0.614; p < 0.001 respectively). Also, percentage transferrin saturation correlated significantly with GGT only, in the treatment-naïve group (r = 0.667; p < 0.001), and ALT and GGT in the treatment group (r = 0.252; p = 0.030 and r = 0.646; p = 0.014 respectively). See Additional file 1.

### Discussion

In this study, the females constituted 54.7% of the treatment group. Similarly, in the treatment-naïve group, there were more females (52.0%) than males (48.0%). Owiredu et al. [5] also observed that higher prevalence of psychiatric disorders were associated with females than males in Ghana [5]. Moreover in a study by Oyane et al. [21], the female gender was more predisposed to factors of psychiatric disorders [21]. The increased female prevalence observed in the current study can be attributed to the fact that, women are emotionally and psychologically fragile in nature and are known to often internalize and brood over problems compared to men [22].

Consistent with the findings of Ikeda [23], this present study observed significantly higher serum levels of iron and percentage transferrin saturation among the treatment-naïve group compared to the treatment and control groups [23]. Moreover, the lower levels of UIBC observed among the treatment psychiatric group and the significantly lower UIBC recorded for the treatment-naïve group also confirms the findings of Ikeda [23], who observed similar levels among epileptic conditions compared to their control counterpart [23].

In agreement with the fact that the liver is the main storage reserve of iron and is significantly affected by excess iron [8, 24], the treatment-naïve group in the current study presented significantly higher AST and GGT levels compared to the control group. Significantly higher AST/ALT ratio was also associated with the treatment-naïve patients compared to the controls, but the levels were comparable to the treatment group. AST/ALT ratio greater than 2:1 and increased GGT levels have been implicated in liver damage [25].

Numerous studies using experimental hepatic iron overload have identified iron-dependent oxidative damage and associated impairment of membrane-dependent functions of the mitochondria, microsomes, and lysosomes [26, 27]. Thus iron-induced lipid peroxidation occurs in hepatocytes and increases the risk of hepatocellular injury [28, 29]. Moreover, the current study recorded significantly lower log ferritin/AST ratio in both the treatment-naïve and treatment groups compared to the control. These significantly lower ratios among both groups may be attributed to the likelihood of liver dysfunction secondary to iron overload [8, 30].

A previous retrospective review by Feifel and Young [15] reported increased plasma iron levels of greater than 170 μg/dl; transferrin saturation greater than 50% and serum ferritin greater than 450 ng/ml among patients with bipolar disorder [15]. Iron dysregulation and overload have also been implicated in Parkinson’s disease [31], Alzheimer’s disease and dementia [32, 33]. Compared to the control group, both males and females in the treatment group reported relatively higher mean levels of iron, transferrin saturation, ALT, GGT and AST, but lower levels of UIBC in the present study. With the exception of AST, ALT and GGT levels, a similar trend observed among the treatment-naïve group revealed significant differences (p < 0.001) in relation to gender. Abnormal serum ferritin (> 300 μg/l in men and > 120 μg/l in women), and transferrin saturation (> 50%) have been reported by Cutler in a case study among psychiatric patients [34], which is consistent with the findings in the current study.

A significant positive correlation between log_10_ferritin and AST, and log_10_ferritin and GGT was observed in both treatment and treatment-naïve groups. This partially agrees with a previous study by Barut et al. [35] which also reported a positive correlation between ferritin and AST levels. This association has been implicated in certain disease conditions such as liver damage [36], malignancy and infection [37]. Moreover, the current study recorded a significant positive correlation between percentage transferrin saturation and GGT only, in the treatment-naïve group, and both ALT and GGT in the treatment group. The association of ferritin and transferrin saturation with the liver function markers suggests that iron metabolism may be associated with liver damage in mental illness [38].

### Conclusion

Iron overload is common among schizophrenia patients in Ghana, and the iron indices are associated with AST/ALT ratio and log Ferritin:AST ratio within treatment-naïve patients. The response of these biological markers has clinical implication on liver performance; thus a possible future risk of fibrosis, mutagenesis and carcinogenesis. This emerging syndemic among schizophrenia patients in Ghana therefore necessitates baseline and periodic medical assessment of iron indices as standard components in the management plans for psychiatric patients.

## Limitations

The current study could not provide information on other potential confounding factors such as Body Mass Index (BMI), menstruation and diet patterns, and hence should be considered in future investigations” With the smaller sample size obtained in this case–control study, only a temporal relationship between iron overload and liver dysfunctions can be established. A further larger longitudinal study is warranted to validate this syndemic relationship among schizophrenics.

## Supplementary information

**Additional file 1: Table S1.** Partial Pearson correlation between Iron markers and liver function markers among study participants.

## Data Availability

All data generated or analyzed during this study are included in this article and its Additional file.

## Abbreviations

UIBC: unsaturated iron binding capacity
TIBC: total iron binding capacity
AST: aspartate transminase
ALT: alanine transminase
GGT: gamma-glutamyl transferase
