# Supplementary material for: Evaluation of serum iron overload, AST:ALT ratio and log10ferritin:AST ratio among schizophrenia patients in the Kumasi Metropolis, Ghana: a case–control study
Source: BMC Res Notes. 2019 Dec 12;12:802. doi: 10.1186/s13104-019-4847-2 (PMC6909526; doi:10.1186/s13104-019-4847-2)
Supplement: Supplementary file 1 — Additional file 1: Table S1. Partial Pearson correlation between Iron markers and liver function markers among study participants. [file 13104_2019_4847_MOESM1_ESM.docx]

**Table S1: Partial Pearson correlation between Iron markers and liver function markers among study participants**

|  |  | Treatment | |  |  | Treatment-naïve | |  |
| --- | --- | --- | --- | --- | --- | --- | --- | --- |
| Markers |  | AST | ALT | GGT |  | AST | ALT | GGT |
| Iron | r | -0.057 | 0.147 | 0.343 |  | 0.114 | -0.034 | -0.068 |
|  | *p*-value | 0.629 | 0.209 | 0.171 |  | 0.329 | 0.772 | 0.561 |
| UIBC | r | -0.113 | -0.011 | -0.004 |  | 0.152 | -0.087 | -0.101 |
|  | *p*-value | 0.335 | 0.925 | 0.798 |  | 0.192 | 0.457 | 0.097 |
| TIBC | r | -0.123 | -0.100 | -0.253 |  | 0.130 | -0.103 | -0.271 |
|  | *p-*value | 0.293 | 0.392 | 0.442 |  | 0.266 | 0.379 | 0.158 |
| Ferritin | r | 0.343 | -0.059 | 0.502 |  | 0.348 | 0.183 | 0.614 |
|  | *p*-value | 0.003 | 0.613 | 0.001 |  | 0.002 | 0.116 | <0.001 |
| STfR | r | -0.113 | -0.011 | -0.233 |  | 0.152 | -0.087 | -0.210 |
|  | *p*-value | 0.334 | 0.926 | 0.309 |  | 0.193 | 0.457 | 0.334 |
| T. Saturation | r | 0.037 | 0.252 | 0.646 |  | 0.006 | 0.048 | 0.667 |
|  | *p*-value | 0.751 | 0.030 | 0.014 |  | 0.961 | 0.686 | <0.001 |

**UIBC: Unsaturated iron binding capacity; TIBC: Total iron binding capacity; AST: Aspartate aminotransferase; ALT: Alanine aminotransferase; GGT: Gamma-glutamyl transferase. r: correlation coefficient. Age and gender adjusted partial correlation**
